# Editorial: The psychological challenges of respiratory disease

**DOI:** 10.3389/fpsyg.2023.1213963

**Published:** 2023-05-24

**Authors:** Eleonora Volpato, Paolo Banfi, Marieke Verkleij

**Affiliations:** ^1^Dipartimento di Psicologia, Università Cattolica del Sacro Cuore, Milan, Italy; ^2^IRCCS Fondazione Don Carlo Gnocchi, Milan, Italy; ^3^Department of Paediatric Psychology, Amsterdam UMC, Vrije Universiteit Amsterdam, Amsterdam, Netherlands

**Keywords:** chronic respiratory disease, psychological factors, adherence, behavioral change, Long-Term Oxygen Therapy, Non-Invasive Ventilation, health psychology

## 1. Introduction

Over the last few decades, there has been a growing body of literature on the influence that psychological factors can have on chronic respiratory diseases and how these, in turn, might have major implications on the psychological distress and quality of life of those affected. Over time, a strong association between increased symptoms of anxiety and depression and chronic respiratory diseases such as Chronic Obstructive Pulmonary Disease (COPD) (Vieira et al., 2011; Ohayon, 2014; Volpato et al., 2023), asthma (Verkleij et al., 2013, 2017), Primary Ciliary Dyskinesia (PCD) (Verkleij et al., 2021a), or Cystic Fibrosis (CF) (Quittner et al., 2016; Verkleij et al., 2018) has been documented Similarly, a strong relationship has been found between certain respiratory symptoms such as dyspnoea, wheezing or nocturnal problems and the onset of anxiety and depression (Leander et al., 2014). Nevertheless, many patients with chronic respiratory diseases do not meet all the diagnostic criteria for mental illness, but still experience high levels of some of their symptoms (e.g., worry, decreased mood, and loss of interest), difficulties in managing their lifestyle (e.g., disturbed sleep and lack of appetite), and other emotional reactions to their illness (e.g., shame, guilt, loneliness, and lower self-esteem) (Verkleij et al., 2018; Jerpseth et al., 2021; Noij et al., 2023; Volpato et al., 2023). In addition, going along with polypharmacy (Ierodiakonou et al., 2021; Woolford et al., 2021), quitting smoking, using Long-Term Oxygen Therapy (LTOT) and/or Non-Invasive Ventilation (NIV) (Mehrtash et al., 2019; Pierucci et al., 2022) require changes in habits and behavior that may, in turn, contribute to the presence of distress and emotional reactions, with consequent effects on self-perception, adherence and clinical outcomes (Jerpseth et al., 2018; McCormick et al., 2022).

However, it is important to note that, on the one hand, the literature on the psychological implications of respiratory health is often controversial and with little clinical applicability, and on the other hand, there is still too little awareness of these issues on the part of both health professionals and the general population.

The main aim of this Research Topic is to present the most recent advances in research on the psychological factors involved in respiratory diseases, as well as their implications in the health management process.

This opening essay outlines the contributions and ways in which they help us better understand the psychological challenges faced by people with a chronic respiratory disease. Unanswered questions will be highlighted to encourage direction to the focus of new research in the future.

The nine original research articles in this Research Topic address three main themes related to psychological challenges in respiratory disease:

- What are the psychological and cognitive factors involved in the use of medications/devices/medical visits and daily activities?
- What are possible innovative psychological treatments for respiratory diseases?
- Which tools can help identify and prevent mental health symptoms in respiratory diseases?

### 1.1. Active or not active?

A substantial body of studies now demonstrates that pulmonary rehabilitation is effective in reducing respiratory symptoms by providing both educational and self-management components to improve exercise capacity, clinical outcomes, quality of life, and understanding of the disease, as well as involvement and adherence to proposed treatments (Hayton et al., 2013; McCarthy et al., 2015; Volpato et al., 2021). Among the psychological factors recently studied in the respiratory disease world are motivation and the Patient Activation Measure (PAM), which provides insight into a person's readiness and role in the care process (Greene and Hibbard, 2012). As demonstrated by Peters et al., a topic as relevant as it is affecting respiratory diseases such as COPD or asthma, a prerequisite to be able to raise the impact of self-management interventions. These constructs are even more important when thought about adaptation to proposed therapies and interventions such as, for example, NIV or Continuous Positive Airway Pressure (CPAP) (Rapelli, Pietrabissa, Angeli, Bastoni et al.; Rapelli, Pietrabissa, Angeli, Manzoni et al.). Exactly for greater engagement in their care process, Friedman et al. involved patients with CF in the structuring of their Cognitive Behavioral Therapy (CF-CBT), which allowed them to denote among the most stressful factors disease management, disease uncertainty, and financial issues (Verkleij et al., 2021b).

### 1.2. When new meets existing

In light of the recent psychological and cognitive factors studied, there is an urgent need to propose multidisciplinary interventions, including innovative approaches such as Acceptance and Commitment Therapy, studied by Giusti et al., or hypnosis (Anlló et al.) as synergistic components with others, especially in the improvement of anxiety and depression's symptoms. Add to this the importance of considering an albeit useful change of setting, as is the case in the study by Gazzi et al., making use of telepsychology and remote interventions, which can also facilitate caregiver participation.

### 1.3. Toward innovative perspectives and tools

In recent years, moreover, the use of different instruments to detect often non-specific psychological and cognitive factors has become apparent, which is why it is important to converge toward the application of measurement tools that differentiate general symptoms of anxiety or depression from those typically found in various populations with respiratory diseases, as in the study of Farver-Vestergaard et al.. Add to this new challenges such as those posed by the COVID-19 pandemic and the use of safety measures that have changed the way we live, as in the case of the use of humidifier disinfectants (Lee et al.).

## 2. Conclusions

Although the intake and delineation of psychological interventions for respiratory diseases is a complex task, requiring integration and process, all papers in this Research Topic highlight new methods and strategies that can be employed to identify psychological and cognitive factors, as well as their treatment, paying attention to the specificity of the conditions. This allows clinicians and scientists to progressively develop cutting-edge interventions that are attentive to personalization and not only to classificatory diagnosis but also to the management of experiences that are not necessarily to be considered mental illnesses.

## Author contributions

This Research Topic on psychological factors in respiratory diseases was initially proposed and set up and this editorial introduction was led by EV. All the editors worked collaboratively to decide which papers were accepted or rejected, and each manuscript was subject to review by the panel of editors as well as peer reviewers. All the editorial team contributed their thoughts and revisions to help craft the published document.
